# Bioinspired Honeycomb-Structured Nanofibrous Membranes with High Transparency and Excellent Breathability for High-Efficiency PM_0.3_ Capture

**DOI:** 10.3390/nano16150941

**Published:** 2026-07-30

**Authors:** Yuan Tian, Xinmiao Wang, Jinhui Wu, Jiancheng Qi

**Affiliations:** Systems Engineering Institute, Academy of Military Sciences, People’s Liberation Army, Tianjin 300161, China; tianyuan2022ty@163.com (Y.T.); wang_xinmiao99@126.com (X.W.)

**Keywords:** particulate matters pollution, air filtration materials, honeycomb-structured, high transparency, high air permeability

## Abstract

Particulate matter (PM) pollution has become a major public health concern. Particularly, PM_0.3_ in the air can cause significant damage to the human respiratory system. However, traditional air filtration materials, due to their limited protective functions, are facing challenges such as poor environmental adaptability, low transparency, and difficulties in balancing filtration efficiency with pressure drop. Inspired by the honeycomb structures and transparent dragonfly wings, this study successfully fabricated a bioinspired honeycomb-structured nanofibrous membranes (NFMs) using template-assisted electrospinning. By optimizing the mesh size of receiver, a directional distribution of the electric field was established on the receiver, promoting the simultaneous concentrated ordered stacking and sparse random orientation of fine-diameter nanofibers. This synergistic strategy of structural optimization and electric field modulation enables NFMs to achieve an optimal balance between filtration efficiency, pressure drop, environmental adaptability and transparency. Utilizing filtration mechanisms involving Brownian diffusion, electrostatic adsorption and physical interception, the honeycomb-structured NFMs achieved a filtration efficiency of over 98.51% for PM_0.3_, with a pressure drop of only 34 Pa, whilst maintaining high transparency (85%) and high air permeability (130.8 mm/s). This bioinspired honeycomb-structured NFMs demonstrates broad application prospects in the field of air purification and offers novel insights for the development of multifunctional air filtration materials.

## 1. Introduction

In recent years, excessive urban development and human activities have further exacerbated air pollution, posing serious challenges to human health and environmental sustainability [1,2]. According to figures reported by the World Health Organization, air pollution has been causing 7 million premature deaths each year, accounting for 10% of global deaths [3]. Particles with a particle size less than 0.3 μm (PM_0.3_) in air pollutants are especially prone to carrying various bacteria and viruses, which will invade human tissues, organs, and blood through the respiratory system, leading to various respiratory and cardiovascular diseases [4,5,6]. In addition, infectious respiratory diseases, such as influenza and the outbreak of novel coronavirus, will also pose a huge threat to human health [7]. At present, the prevention and control measures for air pollution and infectious respiratory diseases are divided into three categories, mainly including reducing pollutant emissions, cutting off the transmission routes of pollutants, and wearing protective equipment. Regardless of the prevention and control measures, high-performance air filtration materials are required as the foundation. Therefore, developing air filtration materials with efficient protective performance is of great significance for protecting human health and promoting green development of the environment. The filtration mechanism of air filtration materials for PMs can be mainly divided into five types, including gravity deposition, inertial action, physical interception, electrostatic adsorption, and Brownian diffusion [8,9,10]. For the highly hazardous PM_0.3_, air filtration materials mainly function through electrostatic adsorption and Brownian diffusion mechanisms [1,11]. Common air filtration materials mainly include electrospun melt blown non-woven fabric and electrospun NFMs [12,13,14]. Surgical masks and N95 masks are currently popular in the market as polarizable non-woven air filters [15]. However, due to the large fiber diameter, the filtration performance of these masks for ultrafine particles is difficult to meet the human demand for high-performance air filters. Furthermore, the multi-layer structure of melt blown non-woven masks will increase air resistance and reduce comfort. Electrospun NFMs have attracted widespread interests among researchers in the prevention and control of air pollutants due to their high porosity, small pore size, high specific surface area, and controllable fiber diameter [12,16,17,18].

Traditional electrospun air filtration materials often suffer from low filtration efficiency and poor pressure drop, making it difficult to achieve a balance between high efficiency and long lifespan [19]. In order to further improve the filtration performance of electrospun air filtration materials, researchers have developed various NFMs with special microstructures by changing electrospinning parameters, optimizing the composition and physicochemical properties of electrospinning solution, mainly including bead, tree branch, and bimodal structure air filtration materials [20,21]. The bead-like structure is beneficial for expanding the distance between nanofibers, optimizing the stacking structure of nanofibers, and further reducing pressure drop [22]. For example, Li et al. successfully embedded polystyrene microspheres into PA6 nanofibers through gas jet spinning, and prepared a PA6/PS NFMs with a “bead chain” shape, achieving outstanding air filtration effect [23]. The tree branch structure air filtration material is composed of coarse fibers and branched fibers. Coarse fibers as the main skeleton are beneficial for improving mechanical properties and reducing pressure drop. Its branch fibers mainly refine the pore size, thereby achieving excellent filtration performance and lower pressure drop. The structure of bimodal structure air filtration material is similar to that of tree branch structure, both consisting of coarse fibers and fine diameter fibers, thereby achieving efficient filtration and low pressure drop [24]. For example, Zheng et al. increased the conductivity of electrospinning solution, which promoted the jet to split and further stretch into bimodal fibers in the electrostatic field, demonstrating excellent particle capture ability [25]. In addition, Xu et al. used parallel spinning to conjugate adjacent PLA nanofibers into micrometer sized fibers, forming a unique bimodal micro nano structure in the electrospun PLA NFMs, thereby enhancing the slip effect of PLA NFMs and significantly reducing pressure drop [10]. The above research results indicate that increasing the distance between fibers and refining pore size through microstructure regulation is beneficial for achieving a considerable balance between filtration efficiency and pressure drop.

In recent years, human production activities have gradually become more diversified, and the protective effect of protective equipment in complex environments has become increasingly important. Especially in high humidity environments, the filtration performance of air filtration materials is greatly affected by humidity. At the same time, after the widespread outbreak of COVID-19, the demand of the masses and medical staff for personalized health protection is growing, which urgently needs the support of new multi-function filter media technology. To address these challenges, Ding et al. prepared a nano mesh fiber membrane through non solvent induced phase separation, achieving efficient filtration performance (with a removal efficiency of 99.8% for PM_0.3_ at pressure drop below 40 Pa), ultra-thin thickness (approximately 800 nm), and 83% high transparency [1]. In addition, based on the “single fiber filtration theory”, Chen et al. prepared transparent screens with a fiber diameter of about 70 nm through electrospinning and polyfluoroalkyl vapor deposition processes, achieving good transparency (~80%) and high filtration efficiency (99.59%) [26]. Afterwards, in order to further improve the comprehensive performance of transparent air filtration materials, Chen et al. successfully constructed a biomimetic transparent air filter material with high transparency (>70.0%), excellent filtration efficiency (PM_0.3_ and PM_1.0_ particles both >99.5%), low pressure drop (<80.0 Pa), and excellent mechanical strength (tensile strength 5.3 MPa, elongation at break 232%) using dual nozzle electrospinning and spatially controlled electric field deposition technology [27]. These research results indicate that by refining the fiber diameter and reducing the thickness of the fiber membrane, high transparency can be achieved while maintaining good filtration efficiency. However, thinner air filtration materials may have issues with low lifespan and susceptibility to damage in practical applications. Thicker small-diameter NFMs will lead to low transparency and poor pressure drop. Therefore, whether it is personal protective equipment or screens in residential environments, they still face difficulties such as insufficient filtration efficiency, poor pressure drop, inferior applicability, and low transparency in complex environments (high humidity).

**Figure 1:**
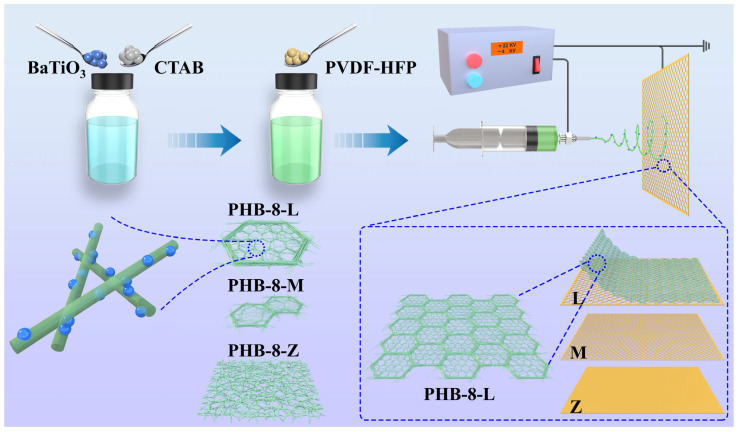
Flowchart of the preparation process for bioinspired honeycomb-structured NFMs.

In this study, we reported a simple, low-cost, and scalable template-assisted electrospinning method for efficiently preparing transparent NFMs with biomimetic honeycomb structures (Figure 1). Macroscopically, the NFMs exhibited a multi-level structure comprising large-scale honeycomb structures containing numerous small-scale honeycombs, bearing a striking resemblance to the structure of a dragonfly’s wings. On a microscopic level, fine-diameter nanofibers formed a honeycomb skeleton through dense and orderly distribution. The sparse fine-diameter nanofibers in the center of honeycomb grid were randomly oriented, providing channels for light and gas to pass through while refining the aperture. This unique structure of NFMs achieved a filtration efficiency of over 98.51% for PM_0.3_ at a wind speed of 32 L/min, while maintaining ultra-low pressure drop (only 34 Pa), high transparency (~85%), and high breathability (130.8 mm/s). In addition, the incorporation of BaTiO_3_ nanoparticles with a high dielectric constant, into NFMs as an electret additive significantly enhanced the charge storage capacity of NFMs, thereby enabling them to maintain a high PM_0.3_ capture efficiency even after 60 filtration cycles. It is worth noting that after working continuously for 24 h in a high humidity environment of 90%, these honeycomb-structured NFMs still maintained high PM_0.3_ filtration performance (filtration efficiency and pressure drop of 98.44% and 68 Pa, respectively), demonstrating satisfactory environmental adaptability. Therefore, this study successfully overcomes the difficult problem of balancing the high efficiency of traditional air filtration materials in filtering ultrafine particles, low pressure drop, high breathability, and high optical transmittance. These biomimetic honeycomb-structured NFMs provide a scalable and multifunctional dual personal protective platform to resist particulate matter pollution, and has broad application prospects in indoor air purification, personal respiratory protection, and transparent medical dressings.

## 2. Experimental Details

### 2.1. Materials

Barium titanate (BaTiO_3_) particles with a purity greater than 99.9% and a particle size of less than 100 nm, N,N-dimethylformamide (DMF) with a purity of over 99.9%, and hexadecyltrimethylammonium bromide (CTAB) with a purity of over 99% were all purchased from Shanghai Aladdin Biochemical Technology Co., Ltd. (Shanghai, China). Polyvinylidene fluoride-co-hexafluoropropylene (PVDF-HFP) with a weight-average molecular weight of 455,000 was purchased from Shanghai Mcline Biochemical Technology Co., Ltd. (Shanghai, China). Stainless steel sheet (SS316) with a thickness of 1 mm was purchased from Zhejiang Taobao Network Co., Ltd. (Hangzhou, China). Polyester mosquito netting as a substrate, featuring a typical small hexagonal mesh, was purchased from Hangzhou Ruiqi Home Furnishings Co., Ltd. (Hangzhou, China) (Figure S1).

### 2.2. Design an Electrospinning Receiver with a Honeycomb Structure

First, a piece of SS316 stainless steel sheet measuring 20 cm in length and 15 cm in width was cut using a 3015GA high-speed laser cutting machine manufactured by Jinan Hongniu Machinery Equipment Co., Ltd. (Jinan, China). The thickness of SS316 stainless steel was 1 mm. The cutting program was then set via the machine’s built-in computer module, with the honeycomb grid side length and gap between cells set at 4 mm and 0.5 mm respectively. Upon starting the machine, the laser cutter followed the set program to cut the SS316 stainless steel sheet into a flat plate receiver composed of a typical large-sized honeycomb grid (Figure S2). By modifying the honeycomb grid side length to 2 mm whilst keeping the remaining parameters unchanged, a flat plate receiver composed of a medium-sized honeycomb grid was produced (Figure S3). An uncut SS316 stainless steel sheet (20 cm x 15 cm) was used as the solid flat-plate receiver (Figure S4). In fact, depending on the cutting capacity of the laser cutting machine, the side lengths of the honeycomb grid on the receiver were set to 4 mm and 2 mm. The receivers obtained from the above procedures-those with large-sized honeycomb structures, those with medium-sized honeycomb structures, and the receiver without a honeycomb structure-were named L, M and Z respectively.

### 2.3. Preparation of Electrospinning Solution

As shown in Figure 1, 17 mL of DMF solvent was weighed out using a graduated cylinder and poured into a 25 mL solution bottle. The weight of the DMF solvent was 15.47 g. In order to enhance the electret effect of NFMs and improve its electrostatic adsorption capacity, 1.69 g of BaTiO_3_ nanoparticles were added to a solution bottle containing DMF. Due to the amphiphilic structure of CTAB (long-chain alkyl and hydrophilic groups), 0.04 g of CTAB was added to the DMF solution to improve the compatibility of BaTiO_3_ in the organic solvent, thereby enhancing the adhesion between BaTiO_3_ nanoparticles and nanofibers. To ensure uniform mixing of the CTAB and BaTiO_3_ nanoparticles with the DMF solvent, the solution bottle was placed on a magnetic stirrer and stirred at 400 rpm at room temperature for 30 min. After stirring, 3.97 g of PVDF-HFP granules was weighed and added to the aforementioned solution bottle. The solution bottle was then placed in a magnetic stirrer and stirred continuously at 400 rpm at 70 °C for 10 h, ultimately yielding a PVDF-HFP spinning solution containing approximately 18.8 wt% PVDF-HFP and 8.0 wt% BaTiO_3_ (Table S1).

### 2.4. Preparation of Honeycomb-Structured NFMs

Using a 5 mL syringe, draw up 3 mL of electrospinning solution. After fitting a 19 G spinning needle, mount the syringe onto the SS-X3 electrospinning machine manufactured by Beijing Yongkang Leye Technology Development Co., Ltd. (Beijing, China). A metal plate with a honeycomb-structure, was used as the receiver, with polyester mosquito netting serving as the substrate (Figure S1). The scanning start point was set at 2 cm, with a scanning stroke of 70 mm, a scanning speed of 500 mm/min, a collection distance of 20 cm, and a gel extrusion speed of 0.08 mm/min. During the electrospinning process, a voltage of −4 kV was applied to the collection plate, whilst a voltage of +22 kV was applied to the syringe. The spinning temperature was approximately 15~20°C, and the humidity was approximately 35~40%. After 50 min of spinning, transparent NFMs with a honeycomb-structure, measuring 20 cm in length and 15 cm in width, were obtained and named PHB-8-L (Figure 1). Furthermore, by setting the electrospinning times to 50 min, 110 min and 150 min respectively, PHB-8-L with basis weight of 4.11 g/m^2^, 7.54 g/m^2^ and 9.61 g/m^2^ was obtained. It is worth noting that the mesh size of substrate was considerably smaller than that of the receiver. Consequently, during the electrospinning process, small honeycomb structures also form in areas of the receiver where the electric field strength is weaker, due to the oriented deposition of nanofibers onto the substrate. Subsequently, by varying the mesh size of the collection plate mesh and using the aforementioned electrospinning parameters, PHB-8-M and PHB-8-Z were prepared using a medium-sized mesh plate and a solid plate as collectors, respectively. Ultimately, the thicknesses of PHB-8-L, PHB-8-M and PHB-8-Z were 0.030 mm, 0.026 mm and 0.023 mm respectively.

### 2.5. Characterizations

The macroscopic morphology of NFMs was observed using a Nikon SMZ745 trinocular stereomicroscope manufactured by Nikon Optical Instruments Co., Ltd. (Wuxi, China). The microscopic surface morphology of NFMs and the intercepted PMs were observed using an SU8600 ultra-high-resolution field-emission scanning electron microscope manufactured by Hitachi, Ltd. (Tokyo, Japan). The nanofiber diameter was measured using the measurement tool supplied with the SU8600 ultra-high-resolution field-emission scanning electron microscope. The diameter of 45 nanofibers was measured for each sample. A statistical analysis of these 45 nanofibers was then performed using a Gaussian function, and a graph of the diameter distribution was plotted. A JEM-F200 field-emission transmission electron microscope, manufactured by JEOL Ltd. (Tokyo, Japan), was used to observe the distribution of nanoparticles and elements within the fibers. The bonding patterns of the fibers were analyzed using an Escalab 250Xi X-ray photoelectron spectrometer, manufactured by Thermo Fisher Scientific Inc. (Waltham, MA, USA). The X-ray source used in the experiment was the Al Kα line (1361 eV). The crystal structure of PVDF-HFP fibers was analyzed using a D8 FOCUS X-ray diffractometer manufactured by Bruker AXS GmbH (Karlsruhe, Germany), with a scanning angle range of 5~50°. The hydrophobic properties of NFMs were tested using a JC2000D3M contact angle measuring instrument manufactured by Beijing Zhongyi Kexin Technology Co., Ltd. (Beijing, China). The hydrophobic properties of NFMs were primarily reflected by the static water contact angle. The static water contact angle was measured three times for each sample, and the average value was then calculated. During the test, the volume of the droplet was 4 μL. The water vapor transmission rate of NFMs was characterized using a type W-B-121F water vapor transmission rate tester. The test was conducted at a temperature of 38°C and a relative humidity of 90%, with an effective test area of 47.78 cm^2^. The pore size and distribution of NFMs were measured using a BSD-PB membrane pore size analyzer manufactured by Best Instrument Technology Co., Ltd. (Dongguan, China) (Text S2). The air permeability of NFMs was characterized using a YG461H fully automatic air permeability tester, with a test pressure difference set at 100 Pa and a test area of 20 cm^2^. The light transmittance of NFMs was analyzed using a GENESYS 10S UV-Vis spectrophotometer manufactured by Thermo Fisher Scientific Inc. (Waltham, MA, USA), with a test wavelength range of 380~780 nm in the visible spectrum.

### 2.6. Filtration Performance Testing

The ability of electrospun NFMs to trap particles of various sizes is the most important indicator for evaluating the filtration performance. By measuring the air pressure $P_{0}$ and $P_{1}$ before and after the aerosol passes through NFMs, the pressure drop ∆P of NFMs at a given air velocity can be calculated using Equation (1). By measuring the aerosol concentrations $C_{0}$ and $C_{1}$ before and after aerosol passes through NFMs, the filtration efficiency ε at a given air velocity can be calculated using Equation (2) [28]. Some NFMs present high filtration efficiency but also poor pressure drop. In order to comprehensively evaluate the filtration performance of NFMs, it is necessary to consider both the filtration efficiency ε and the pressure drop ∆P. Therefore, the quality factor QF, calculated using Equation (3) on the basis of filtration efficiency and pressure drop, facilitates a scientific analysis of the filtration performance of NFMs [29]. Based on this theory, we conducted a thorough analysis of the filtration performance, using the SX-L1056R1 filtration performance tester manufactured by Suzhou Suxin Environmental Technology Co., Ltd. (Suzhou, China) (Figure S5), with a test area of 100 cm^2^. In order to compare performance with other air filtration materials, the test airflow rate for filtration performance was set primarily at 32 L/min. To ensure the accuracy of the tests, the NaCl aerosol concentration and size distribution were maintained in line with those in the ambient atmosphere (Table S4). Furthermore, an airflow rate of 32 L/min is slightly higher than the normal respiratory airflow of a human being. Therefore, setting the test air flow rate to 32 L/min will help to scientifically assess the ability of NFMs to act as a mask and block contaminants. In order to investigate the environmental adaptability of the NFMs, their filtration performance was tested after it had been exposed to an environment with 90% humidity for 24 h. Furthermore, without any cleaning treatment, cyclic tests were carried out on the NFMs to assess their long-term filtration performance.

(1) $$∆P=P_{1}-P_{0}$$

(2) $$\varepsilon =(1-C_{1}/C_{0})\cdot 100\%$$

(3) $$QF=-\ln\left(1-\varepsilon \right)/∆P\cdot 100\%$$

## 3. Results and Discussion

### 3.1. The Composition and Structure of Honeycomb-Structured NFMs

**Figure 2:**
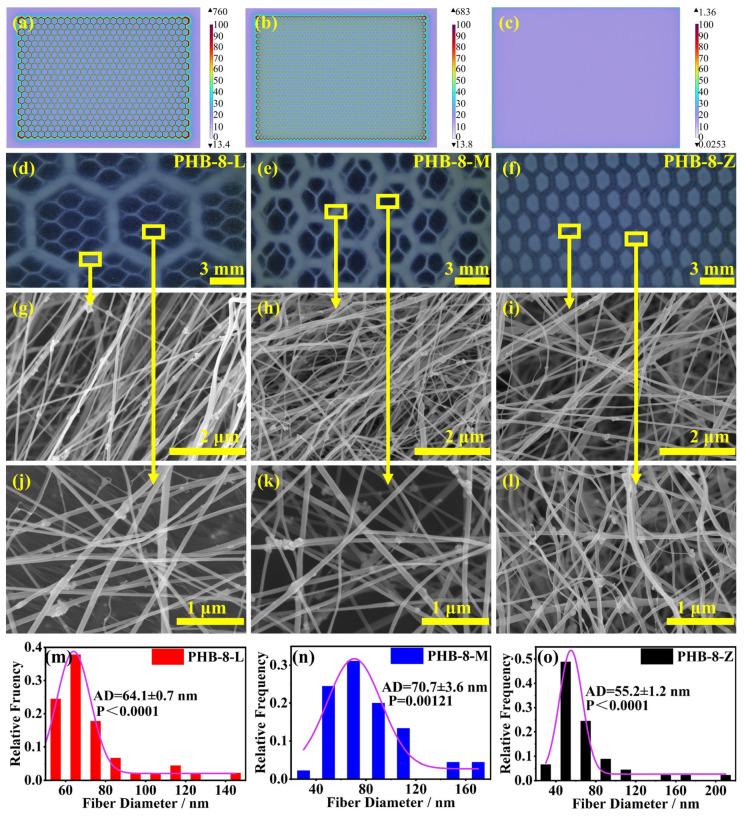
The electrostatic field distribution of the receiver plates, SEM images and fiber diameter distribution of bioinspired honeycomb-structured NFMs: (**a**,**d**,**g**,**j**,**m**) PHB-8-L; (**b**,**e**,**h**,**k**,**n**) PHB-8-M; (**c**,**f**,**i**,**l**,**o**) PHB-8-Z.

In this study, we aimed to construct special morphology NFMs by changing the grid structure of receiver and regulating the electric field distribution on the receiver. The electrostatic field distribution of the needle-back electrode system was investigated through finite element analysis using the COMSOL Multiphysics^®^ software package, specifically its AC/DC Module with the Electrostatics interface (Text S1). As shown in Figure 2a,b, the maximum electric field strengths for the PHB-8-L, PHB-8-M and PHB-8-Z were 760, 683 and 1.36 respectively. The electric field strength on the L and M receiver plates was mainly concentrated on the grid, whilst the electric field strength at the center of the grid is weaker. Compared with the L and M receiver plates, the electric field strength on the Z receiver plate tended to be uniformly distributed (Figure 2c). The electric field strengths distributed across multiple regions on the L and M receiver plates will cause nanofibers to tend to deposit on the mesh of receiver plates. In contrast, only a small number of nanofibers will be present in those regions where the electric field strength is weaker. As shown in Figure 2d, the PHB-8-L prepared by a large grid receiver has a typical honeycomb structure, with small honeycombs distributed in the large grid honeycomb. Compared with PHB-8-L, as the grid size of receiving plate decreases, the honeycomb size of PHB-8-M and PHB-8-Z prepared gradually decreases (Figure 2e,f). In order to effectively enhance the electrostatic adsorption capacity of NFMs, we introduced BaTiO_3_ nanoparticles with high dielectric constant into the electrospinning solution during the preparation process [30]. As shown in Figure 2g–l, a large number of nanoparticles adhere to the surfaces of all the NFMs, possibly introduced BaTiO_3_ nanoparticles. Furthermore, the average fiber diameters of PHB-8-L, PHB-8-M and PHB-8-Z were 64.1 ± 0.7 nm, 70.7 ± 3.6 nm and 55.2 ± 1.2 nm, respectively (Figure 2m–o). The fine diameter of these fibers was very close to the air free path (66 nm), which is conducive to inducing the air slip effect, thereby reducing pressure drop [18,31]. A normality test was conducted on the fiber diameter of each sample. The results showed that all *p*-values were less than 0.05, indicating that the fiber diameters of the three NFMs did not follow a normal distribution (Figure 2m–o). It is worth noting that the nanofibers on PHB-8-L and PHB-8-M grids exhibit dense high orientation (Figure 2g,h), while the nanofibers in the grids exhibit sparse random orientation (Figure 2j,k). This integrated multi zone structural design is similar to the wing structure of dragonflies, providing a large number of channels for light and gas to pass through [27,32]. Compared with the other two types of NFMs, PHB-8-Z exhibited the characteristic of dense and randomly oriented fibers at the microscopic level, which may increase light reflection and air resistance (Figure 2i,l). Furthermore, due to the multi-region aggregation distribution of the nanofibers, the root-mean-square roughness of PHB-8-L, PHB-8-M and PHB-8-Z was 348 nm, 320 nm and 258 nm, respectively (Figure S6).

**Figure 3:**
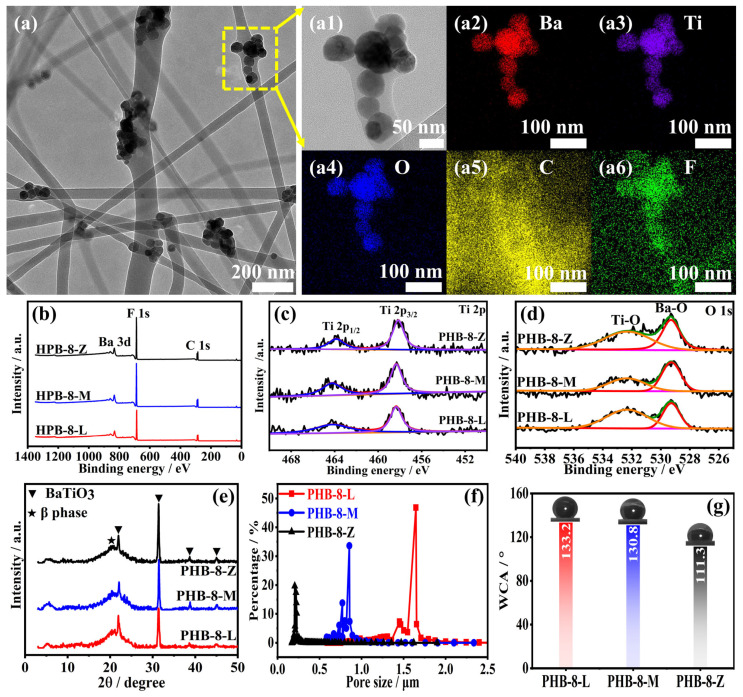
Microstructure of biomimetic honeycomb-structured NFMs: (**a**) TEM images of PHB-8-L; (**a1**–**a6**) TEM images and elemental mapping of PHB-8-L; (**b**) XPS full scan spectra of NFMs; (**c**) XPS high-resolution spectra of Ti element in NFMs; (**d**) XPS high-resolution spectra of O element in NFMs; (**e**) XRD spectra of NFMs; (**f**) pore size and distribution maps of NFMs; (**g**) static water contact angle of NFMs.

To further clarify the composition of nanoparticles adhering to the fiber surface and their bonding with the fibers, we conducted a detailed compositional and structural analysis of all the NFMs using a variety of characterization methods. As shown in Figure 3a, nanoparticles are embedded not only on the fiber surface but also within the fibers. The nanoparticles are firmly bonded to the nanofibers and do not detach easily, which is conducive to the activation of fiber surface and thus improves the ability of nanofibers to adsorb pollutants (Figure 3(a1)) [33]. The element mapping image shows that the positions of nanoparticles are mainly Ba, Ti, and O elements, indicating that the nanoparticles on fiber surface and inside fiber are BaTiO_3_ (Figure 3(a2–a6)). In addition, the XPS full scan images show that all the NFMs are mainly composed of F and C elements, as the main component is PVDF-HFP (Figure 3b). The signals for Ba, Ti and O elements in the full-scan XPS spectra are relatively weak, as the BaTiO_3_ content is lower and its distribution is relatively sparse compared to PVDF-HFP (Figure 3a). The above results indicate that BaTiO_3_ nanoparticles have been successfully incorporated into all the NFMs via in-situ blending. Under normal circumstances, the mode of interaction between functional additives and nanofibers has a significant impact on the performance of NFMs. By analyzing the high-resolution spectra of Ba and O elements, the bonding mode between BaTiO_3_ nanoparticles and fibers can be explored clearly. In the high-resolution Ti spectra, two distinct characteristic peaks are observed in all the NFMs (Figure 2c). The sharp peak at a binding energy of 456.9 eV is attributed to Ti 2p_1/2_, whilst the broad peak at 462.8 eV is attributed to Ti 2p_3/2_, indicating that Ti primarily bonds with O to form oxides [34]. In the O 1 s spectrum, the signal peaks at 529.3 eV and 532.4 eV are attributed to Ba–O and Ti–O bonds, respectively (Figure 3d). Furthermore, distinct crystal structure characteristic peaks are observed in the XRD patterns of all NFMs (Figure 3e). The shoulder at 2θ = 20.5° is attributed to diffraction from the β-phase crystal plane, indicating that the crystal structure of PVDF-HFP is predominantly of the β-phase. Distinct diffraction peaks are observed at 2θ = 22°, 31.4°, 38.7° and 45° for all the NFMs, corresponding to the (100), (110), (111) and (200) crystal planes of BaTiO_3_ nanoparticles, respectively [34]. Therefore, BaTiO_3_ nanoparticles were successfully introduced into all the NFMs without changing the polarizability of BaTiO_3_, which is beneficial for enhancing the electrostatic adsorption capacity of NFMs for particles [35].

The filtration performance of air filtration materials is closely related to the pore size. Generally speaking, a larger aperture is beneficial for improving breathability and optical transparency but weakens the ability of NFMs to capture inhalable particles. Small aperture is beneficial for filtering inhalable particles, but it can hinder the passage of gas and light, and increase pressure drop. Therefore, only NFMs with moderate pore size can fully demonstrate excellent filtration performance. As shown in Figure 3f, the average pore sizes of PHB-8-L, PHB-8-M, and PHB-8-Z were 1.59 ± 0.64 μm, 0.84 ± 0.50 μm and 0.22 ± 0.45 μm, respectively (Table S3). This is mainly due to the sparse distribution of nanofibers in the grid of PHB-8-L, while they exhibit dense stacking in PHB-8-Z (Figure 2j,l). With the diversification of production activities, humans have put forward higher requirements for the filtration performance of NFMs in complex environments. Currently, most air filtration materials have poor adaptability in high humidity environments, and water vapor in the environment can accelerate static dissipation. As shown in Figure 3g, the static water contact angles of PHB-8-L, PHB-8-M, and PHB-8-Z were 133.2°, 130.8°, and 111.3°, respectively, indicating these NFMs have considerable hydrophobic properties. This difference is attributed to variations in the surface roughness of the NFMs resulting from the distribution of the nanofibers (Figure S6). Hydrophobic performance can not only improve the comfort of air filtration materials as protective masks, but also effectively reduce the impact of environmental humidity on charge stability [6,33].

### 3.2. The Breathability and Optical Transparency of Honeycomb-Structured NFMs

**Figure 4:**
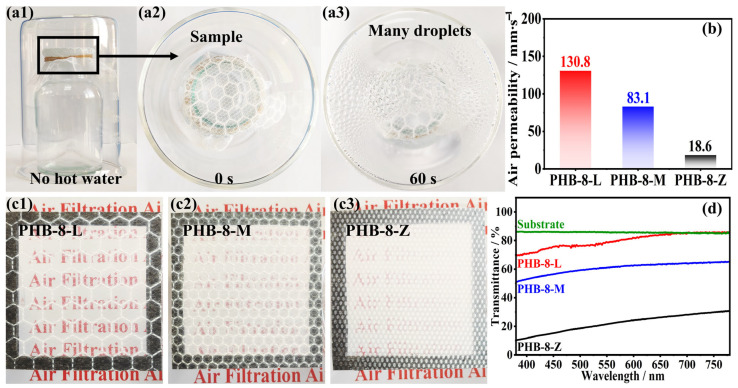
(**a1**–**a3**) Experimental display of water vapor transmission through PHB-8-L, (**b**) air permeability, (**c1**–**c3**) optical images of the three NFMs, (**d**) visible light transmittance.

To fully validate the superior hydrophobicity and breathability of honeycomb-structured NFMs, we devised a simple apparatus for simulating water vapor transmission through the NFMs under high-humidity conditions mimicking mask service environments. As shown in Figure 4(a1,a2), we secured PHB-8-L to the mouth of a solution bottle using rubber bands and then placed a large beaker upside down directly above the bottle. When no hot water is added to the solution bottle, the top of beaker is completely transparent, and the PHB-8-L inside can be clearly seen through the top of beaker (Figure 4(a2)). After adding hot water to the solution bottle for 60 s, it can be clearly observed that a large amount of water vapor condenses into water droplets at the top of beaker (Figure 4(a3)). This phenomenon is caused by the easy passage of water vapor through the NFMs, indicating that the PHB-8-L has outstanding hydrophobic and breathable properties. More importantly, the water vapor transmission rate of PHB-8-L, PHB-8-M and PHB-8-Z under conditions of 38 °C and 90% humidity was 6786 g/(m^2^·24 h), 6382 g/(m^2^·24 h) and 6254 g/(m^2^·24 h), respectively, indicating that PHB-8-L possessed excellent breathability (Figure S7). In order to verify the excellent air permeability of honeycomb-structured NFMs, we conducted air permeability tests on these three types of NFMs. As shown in Figure 4b, under a pressure difference of 100 Pa, the air permeability of PHB-8-L, PHB-8-M, and PHB-8-Z are 130.8 mm/s, 83.1 mm/s, and 18.6 mm/s, respectively, indicating that the air permeability of honeycomb-structured NFMs (PHB-8-L) was seven times that of dense NFMs (PHB-8-Z). It is worth noting that the air permeability of PHB-8-L was much higher than previously reported NFMs, including BaTiO_3_/PLA fiber membrane (77 mm/s), MOF/PLA fiber membrane (49.2 mm/s), and nanofiber/mesh membrane (less than 75 mm/s at a pressure difference of 100 Pa) [30,36,37].

The optical image of PHB-8-L shows that it has excellent transparency performance, and the words behind can be clearly seen through the NFMs (Figure 4(c1)). Compared with PHB-8-L, the transparency performance of the other two NFMs are relatively low, and the following words are basically invisible (Figure 4(c2,c3)). In the visible light wavelength range (380~780 nm), the visible light transmittance of PHB-8-L was significantly higher than the other two NFMs (Figure 4d). At a wavelength of 780 nm, the visible light transmittance of PHB-8-L, PHB-8-M, and PHB-8-Z was 85%, 66%, and 31%, respectively. Furthermore, the visible light transmittance of pure PHB-8-L at 780 nm was essentially consistent with that of the substrate. These results indicated that PHB-8-L possessed excellent transparency and showed promise as a transparent screen window to efficiently block outdoor pollutants from entering indoors. The excellent transparency performance of PHB-8-L is attributed to the sparse random orientation structure of nanofibers in the honeycomb grid, which provides a large number of channels for light to pass through (Figure S8).

### 3.3. Filtration Performance of Honeycomb-Structured NFMs

**Figure 5:**
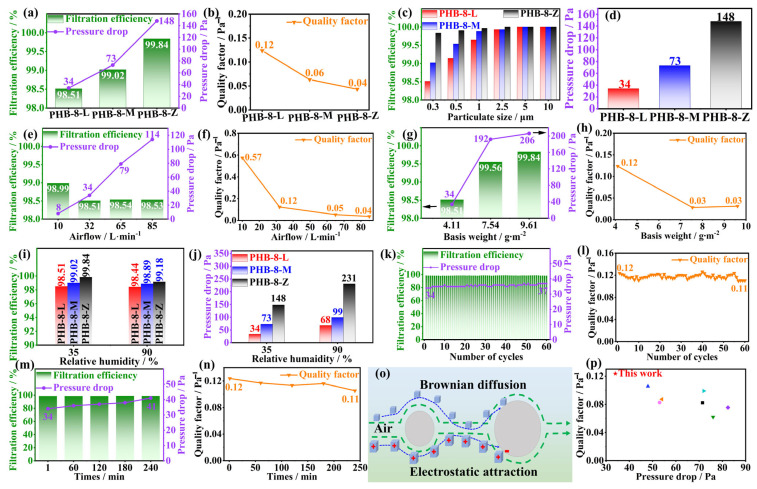
Filtration performance of bioinspired honeycomb-structured NFMs: (**a**,**b**) filtration performance of the three NFMs against PM_0.3_ at 35% humidity and an air velocity of 32 L/min, (**c**,**d**) filtration performance of the three NFMs against different PMs at 35% humidity and an air velocity of 32 L/min, (**e**,**f**) filtration performance of PHB-8-L against PM_0.3_ under different air flow rates, (**g**,**h**) the relationship between the filtration performance of PHB-8-L against PM_0.3_ and basis weight, (**i**,**j**) filtration performance of the three NFMs against PM_0.3_ under different humidity conditions, (**k**,**l**) results of cyclic testing of the filtration performance of PHB-8-L against PM_0.3_, (**m**,**n**) filtration performance of the PHB-8-L against PM_0.3_ after 240 min of continuous operation, (**o**) diagram illustrating the air slip effect and filtration mechanism of fine-diameter fibers, (**p**) comparison of the filtration performance of PHB-8-L with other NFMs [6,11,28,45,46,47,48].

In order to accurately evaluate the filtration performance of NFMs, we conducted comprehensive filtration performance tests on the three types of NFMs prepared using a filtration performance tester (Figure S5). As shown in Figure 5a, the filtration efficiencies of PHB-8-L, PHB-8-M, and PHB-8-Z for PM_0.3_ under a wind speed of 32 L/min were 98.51%, 99.02%, and 99.84%, respectively, with pressure drop of 34 Pa, 73 Pa, and 148 Pa, respectively. The main reason for the difference in filtration performance is the different distribution patterns of nanofibers in the three NFMs (Figure 2d–l). As shown in Figure 5b, the quality factors of PHB-8-L, PHB-8-M, and PHB-8-Z are 0.12 Pa^−1^, 0.06 Pa^−1^, and 0.04 Pa^−1^, respectively. In addition, the quality factor of PHB-0-L without barium titanate was only 0.06 Pa^−1^, which was significantly lower than that of PHB-8-L (0.12 Pa^−1^), indicating that barium titanate enhanced the electrostatic adsorption capacity of NFMs for PM_0.3_ (Figure S9). The above results indicate that PHB-8-L possessed excellent filtration performance for PM_0.3_, with a significantly higher quality factor than the other two NFMs, and a lower pressure drop than the resistance during normal breathing [5]. The types of aerosols in the air are complex, mainly including particles of various sizes and bacteria adsorbed on the particles. Therefore, the filtration efficiency of NFMs for other particle sizes is also an important indicator for evaluating whether NFMs can be used as air filtration materials. Figure 5c,d show the filtration performance of three types of NFMs for various particle sizes (PM_0.3_, PM_0.5_, PM_1_, PM_2.5_, and PM_10_). As the particle size increases, the filtration efficiency of the three types of NFMs for PMs gradually increases. For another type of PMs with more serious harm (PM_2.5_), the filtration efficiency of PHB-8-L, PHB-8-M, and PHB-8-Z for PM_2.5_ is greater than 99.9%, with pressure drops of 34 Pa, 73 Pa, and 148 Pa, respectively.

When NFMs are used as screens or masks to block outdoor polluted air, they face significant changes in wind speed. Therefore, we tested the relationship between filtration performance of PHB-8-L for PM_0.3_ and wind speed, as shown in Figure 5e. When the wind speed is 10 L/min (close to the respiratory rate of an ordinary person), the filtration efficiency is 98.99%, and the pressure drop is extremely low (only 8 Pa) [24]. When the wind speed increases to 32 L/min, there is little change in filtration efficiency and pressure drop, maintaining a quality factor of up to 0.12 Pa^−1^ (Figure 5f). Even when the wind speed increases to 85 L/min, the decrease in filtration efficiency is only 0.46% (negligible), and the pressure drop increases to 114 Pa. Consequently, the pressure drop at a wind speed of 85 L/min is still significantly lower than that of the sandwich-structure NFMs (~180 Pa), demonstrating excellent respiratory comfort [38]. Air filtration materials have played an important role in protecting human health and purifying the air. Nowadays, air filtration materials are indispensable in a wide range of human production activities, and the widespread use of air filtration materials will exacerbate energy consumption. We prepared PHB-8-L with different basis weights by extending the electrospinning time and tested the filtration performance of NFMs with different basis weights. As shown in Figure 5g,h, the PHB-8-L, at a low basis weight (4.11 g/m^2^), exhibited a filtration efficiency of 98.51%, a pressure drop of 34 Pa, and a quality factor of 0.12 Pa^−1^, indicating that the ability of PHB-8-L to filter PMs is significantly more efficient than previously reported maize-based air filters, superhydrophobic/oleophobic FR-COC-QAS NFMs, PEI/PVDF-HFP/FAS NFMs and rod-ribbon interwoven NFMs, which is beneficial for the sustainable development of air filtration materials [5,39,40,41]. In addition, as the basis weight of PHB-8-L increases, the filtration efficiency, pressure drop, and quality factor all show non curvilinear growth, which may be related to the distribution of nanofibers in different regions.

The environmental humidity of air filtration materials varies significantly in different usage scenarios or seasons. Traditional air filtration materials are prone to charge dissipation and pore blockage in high humidity environments, leading to a decrease in filtration performance [42]. Therefore, we placed three types of NFMs in a sealed environment with 90% humidity for 24 h to test their filtration performance. When the humidity increased from 35% to 90%, the filtration efficiency of PHB-8-L and PHB-8-M remained basically unchanged (Figure 5i), and the change in pressure drop was approximately 30 Pa (Figure 5j), mainly due to their excellent hydrophobic properties and high breathability. Owing to its dense fiber structure and insufficient hydrophobicity, PHB-8-Z experienced a decrease in filtration efficiency and an increase in pressure drop to 231 Pa under 90% humidity conditions. It is worth noting that the filtration performance of PHB-8-L under 90% conditions is significantly higher than that of the N95 mask (92.98%), demonstrating excellent environmental adaptability [43]. Furthermore, service life is one of the important indicators for evaluating filtration performance of air materials [44]. As shown in Figure 5k,l, after 60 consecutive test cycles without cleaning (each test cycle lasts approximately 3 min), the filtration efficiency of PHB-8-L for PM_0.3_ remained basically unchanged (with a decrease of only 0.2%), the pressure drop slightly increased from 34 to 37 Pa, and the quality factor slightly decreased from 0.12 to 0.11 Pa^−1^. In addition, we tested the filtration performance of PHB-8-L for PM_0.3_ every 60 min. As shown in Figure 5m, after continuous operation for 240 min, the filtration efficiency of PHB-8-L remained basically unchanged (with a variation of about 0.1%), and the pressure drop slightly increased from 34 to 41 Pa. What is more, the quality factor decreased from 0.12 to 0.11 Pa^−1^ still maintaining a high-quality factor (Figure 5n). These results indicate that PHB-8-L exhibits considerable durability and excellent filtration stability. The slight increase in pressure drop following continuous operation of NFMs is attributed to the adsorption of NaCl aerosol on the membrane surface owing to prolonged filtration, which causes blockage of the nanofiber pores [24].

**Figure 6:**
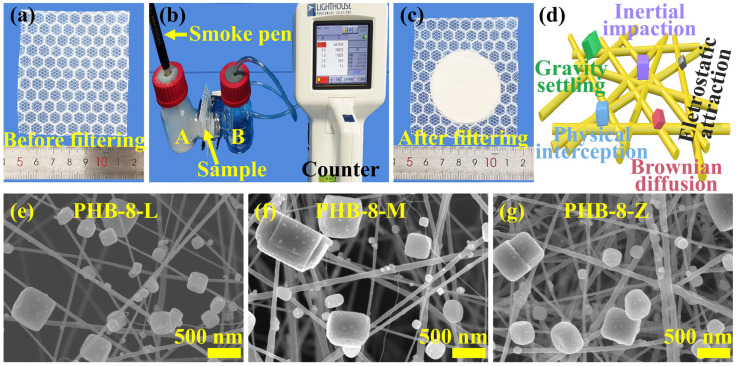
(**a**–**c**) Smoke filtration device diagram of honeycomb-structure NFMs, (**d**) Schematic diagram illustrating the filtration mechanism of fiber-based air filtration materials, (**e**) SEM image of the PHB-8-L after filtering NaCl aerosol, (**f**) SEM image of the PHB-8-M after filtering NaCl aerosol, (**g**) SEM image of the PHB-8-Z after filtering NaCl aerosol.

In order to evaluate the ability of honeycomb-structured NFMs as a screen window to prevent outdoor pollutants from entering the indoor environment. We conducted an experiment as shown in Figure 6 to analyze the practical application effect of honeycomb-structured NFMs. Figure 6a shows the PHB-8-L with a width of 6.5 cm, which was attached to a polyester substrate. As shown in Figure 6b, the left A glass bottle simulated an outdoor environment, and a smoking pen was placed in bottle A as a pollution source. The B glass bottle on the right simulated a clean indoor environment. The PHB-8-L attached to a polyester substrate was placed between bottle A and bottle B (Figure 6b). To simulate a ventilated environment and quantitatively analyze the smoke-filtering performance of NFMs, we connected an aerosol particle counter to bottle B. After igniting the smoking pen, it can be clearly seen that the smoking pen continuously produced a lot of smoke in the A glass bottle, and the visibility is extremely low. The cleanliness of B glass bottle was very high, and no smoke could be seen. More importantly, the concentration of PM_0.3_ in bottle B was only 747,918, which was essentially the same as the concentration of PM_0.3_ found in fresh air in the natural environment (Figure S10). Compared with the PHB-8-L before smoke filtration (Figure 6a), the surface of PHB-8-L after smoke filtration contains a large number of pollutants (Figure 6c), demonstrating excellent smoke filtration ability. In general, the filtration mechanism of air filtration materials for PMs mainly includes gravity settling, inertial impaction, physical interception, electrostatic adsorption, and Brownian diffusion (Figure 6d). In order to investigate the filtration mechanism of NFMs, we conducted SEM analysis on three types of NFMs after filtering NaCl aerosols. As shown in Figure 6e, PHB-8-L intercepted large particle size NaCl aerosol through physical interception. Due to the refinement of fibers and activation of fiber surfaces, PHB-8-L captured PMs with a diameter below 300 nm through electrostatic adsorption and Brownian diffusion (Figure 5o), including PM_0.3_ and PM_0.1_ [33]. In addition, at a flow rate of 32 L/min, the PHB-8-L captured approximately 35 NaCl aerosol particles (Figure 6e). Of these, five had a particle size greater than 0.3 micrometers, whilst the remainder were all smaller than 0.3 micrometers. If we consider only the mechanisms of physical interception, electrostatic adsorption and Brownian diffusion, the efficiency of physical interception was approximately 14.3%, whilst electrostatic adsorption and Brownian diffusion together accounted for 85.7%. Therefore, we may roughly conclude that PHB-8-L captured PM_0.3_ primarily through the mechanisms of electrostatic adsorption and Brownian diffusion. Although the other two NFMs also remove PMs via the same filtration mechanism, their relatively smaller pore sizes and higher fiber packing density result in poor pressure drop (Figure 6f,g). More importantly, compared with various air filtration materials reported in the literature, the honeycomb-structured PHB-8-L exhibited lower pressure drop (34 Pa) and a higher quality factor (0.12 Pa^−1^), making it suitable for use as a transparent screen window for the efficient filtration of PMs (Figure 5p and Table S2) [6,11,28,45,46,47,48].

## 4. Conclusions

In summary, we have successfully prepared transparent honeycomb-structured NFMs through template-assisted electrospinning. By adjusting the grid size of the flat panel receiver, the electric field distribution on flat panel receiver was further changed, resulting in both dense ordered distribution and sparse random orientation of the fine-diameter fibers. On a microscopic level, the sparse random orientation of fine-diameter fibers provides a large number of channels for the passage of light and gas. Thanks to the porous structure of NFMs and the activation of fiber surface, the honeycomb-structured NFMs significantly enhances their ability to capture ultrafine PMs. The results showed that PHB-8-L with honeycomb-structure exhibited high transparency (~85%), long service life, good environmental adaptability, and a filtration efficiency of up to 98.51% for PM_0.3_, while the pressure drop was only 34 Pa. Consequently, the optimized honeycomb-structured NFMs, whilst ensuring filtration performance, possess multifunctional characteristics and holds promise for the removal of ultrafine NaCl aerosols from the air, offering a novel air filtration material for air purification.

## Data Availability

Data will be made available on request.

## Supplementary Materials

The following supporting information can be downloaded at: https://www.mdpi.com/article/10.3390/nano16150941/s1, Table S1: The concentrations of all components in the spinning solution; Table S2: Comparison table of the filtration performance of different types of air filter media; Table S3: The pore size results for the NFMs; Table S4: The NaCl aerosol concentration and size distribution; Figure S1: Photograph of polyester mosquito netting as a substrate; Figure S2: Schematic diagram of a receiver with a large-scale honeycomb structure; Figure S3: Schematic diagram of a receiver with a medium-sized honeycomb structure; Figure S4: Schematic diagram of a receiver without a honeycomb structure; Figure S5: Photograph of the SX-L1056R1 filter performance test bench; Figure S6: Three-dimensional atomic force microscopy images of NFMs: (a) PHB-8-L, (b) PHB-8-M, (c) PHB-8-Z; Figure S7: The WVTR of NFMs; Figure S8: Schematic diagram of the optical structure of the PHB-8-L NFMs; Figure S9: A comparison of the quality factors of PHB-0-L (without barium titanate) and PHB-8-L; Figure S10: Photographs showing particulate matter concentrations before and after filtration of smoke using the PHB-8-L: (a) Before filtering; (b) After filtering; Text S1: The electrostatic field distribution of the receiver plates; Text S2: The specific test procedure of the bubble point method.
