# Supplementary material for: Bioinspired Honeycomb-Structured Nanofibrous Membranes with High Transparency and Excellent Breathability for High-Efficiency PM0.3 Capture
Source: Nanomaterials (Basel). 2026 Jul 30;16(15):941. doi: 10.3390/nano16150941 (PMC13467724; doi:10.3390/nano16150941)
Supplement: Supplementary file 1 [file nanomaterials-16-00941-s001.zip › nanomaterials-4397128-supplementary.pdf]

Support Information

Bioinspired Honeycomb-Structured Nanofibrous Membranes with High Transparency and Excellent Breathability for High-Efficiency $PM_{0.3}$ Capture

Yuan Tian, Xinmiao Wang, Jinhui Wu * and Jiancheng Qi *

Systems Engineering Institute, Academy of Military Sciences, People's Liberation Army, Tianjin 300161, China;
tianyuan2022ty@163.com (Y.T.); wang_xinmiao99@126.com (X.W.)
* Correspondence: wujh@npec.org.cn (J.W.); qijch@npec.org.cn (J.Q.)

Table S1. The concentrations of all components in the spinning solution.

Sample	The spinning solution
DMF solvent content (wt%)	73.1
PVDF-HFP content (wt%)	18.8
$BaTiO_3$ content (wt%)	8.0
CTAB content (wt%)	0.2

Table S2. Comparison table of the filtration performance of different types of air filter media.

Sample	Presssure drop / Pa	Quality factor / Pa^{-1}	Reference
Biomimetic nanofibrous aerogels	71.48	0.08	[46]
Self-powered wood aerogels	53	0.08	[47]
PVDF-HFP@PA66/H	48	0.11	[48]
Noodle-like curly NFMs	76	0.06	[49]
PLA NFMs	82.4	0.08	[28]
Polyvinylidene fluoride/polysulfone NFMs	54	0.09	[50]
PEG-modified biomass-based polyester and forsythin	72	0.10	[11]
PHB-8-L	34	0.12	This work

Table S3. The pore size results for the NFMs.

Sample	PHB-8-L	PHB-8-M	PHB-8-Z
The average pore sizes	1.59±0.64 μm	0.84±0.50 μm	0.22±0.45 μm

Table S4. The NaCl aerosol concentration and size distribution.

The NaCl aerosol size distribution	The NaCl aerosol concentration
0.3 μm	280374
0.5 μm	192486
1.0 μm	69203
3.0 μm	16595
5.0 μm	3055
10.0 μm	977

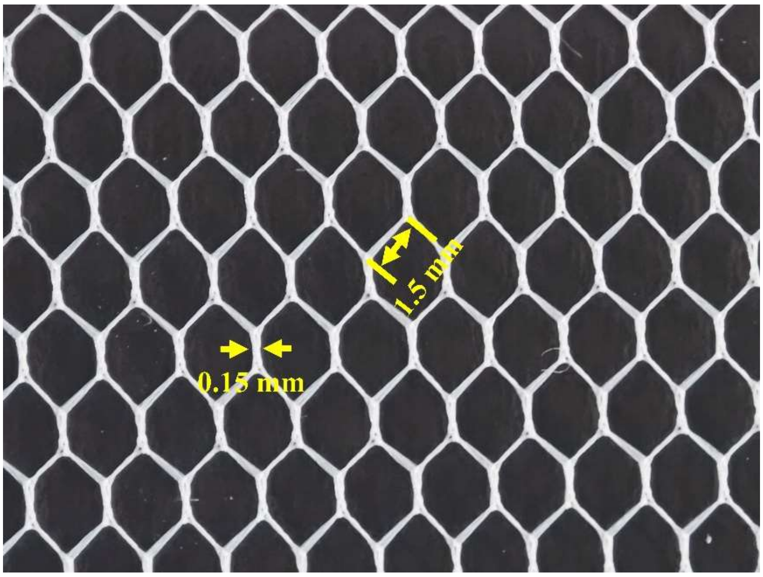

Figure S1. Photograph of polyester mosquito netting as a substrate.

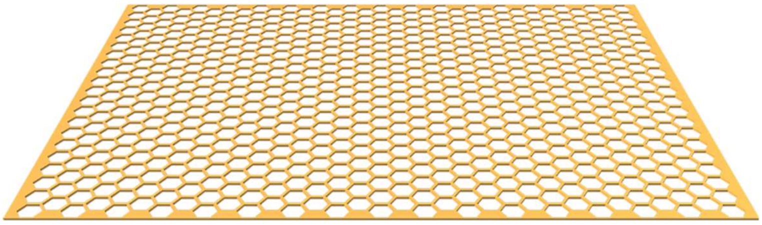

Figure S2. Schematic diagram of a receiver with a large-scale honeycomb structure.

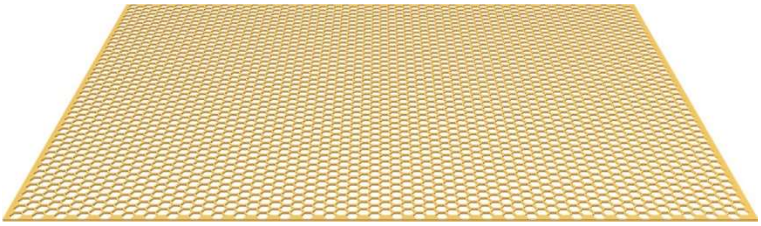

Figure S3. Schematic diagram of a receiver with a medium-sized honeycomb structure.

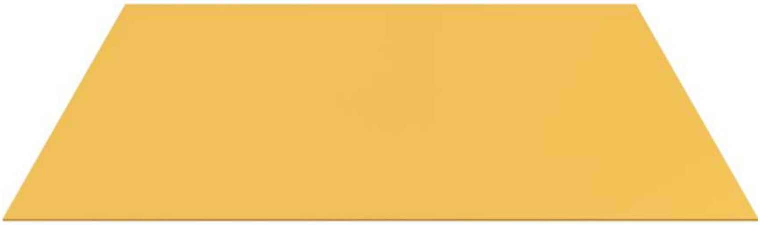

Figure S4. Schematic diagram of a receiver without a honeycomb structure.

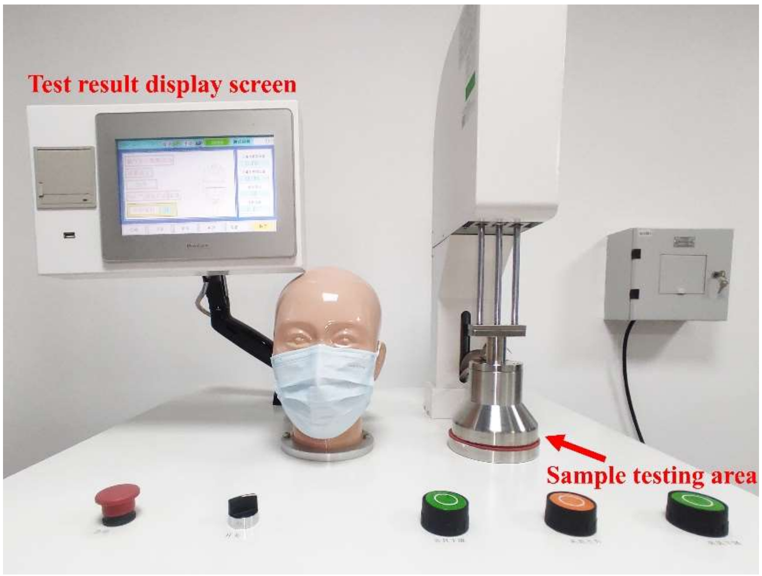

Figure S5. Photograph of the SX-L1056R1 filter performance test bench.

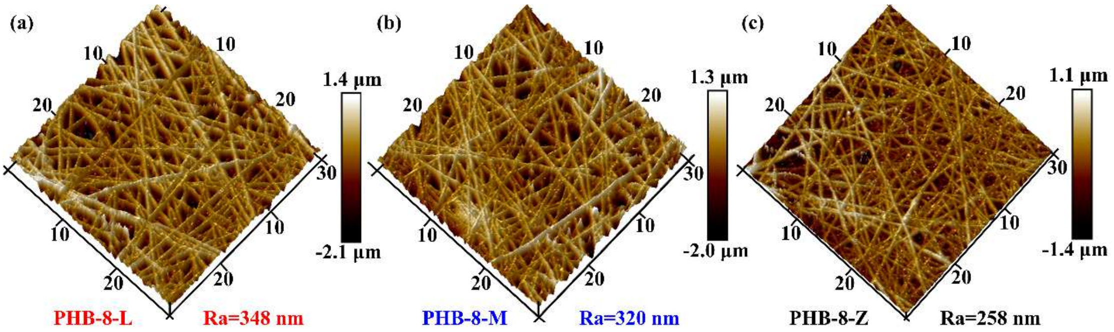

Figure S6. Three-dimensional atomic force microscopy images of NFMs: (a) PHB-8-L, (b) PHB-8-M, (c) PHB-8-Z.

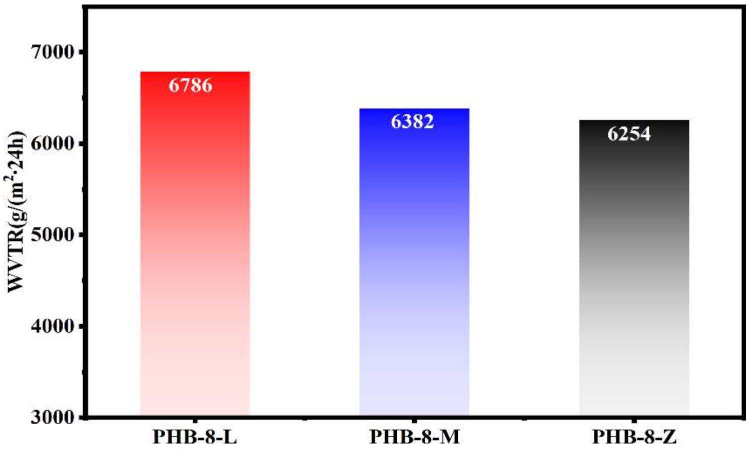

Figure S7. The WVTR of NFMs.

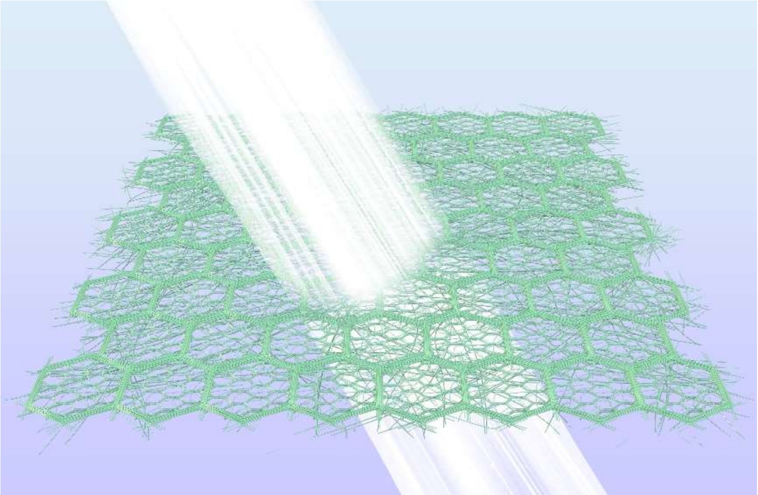

Figure S8. Schematic diagram of the optical structure of the PHB-8-L NFMs.

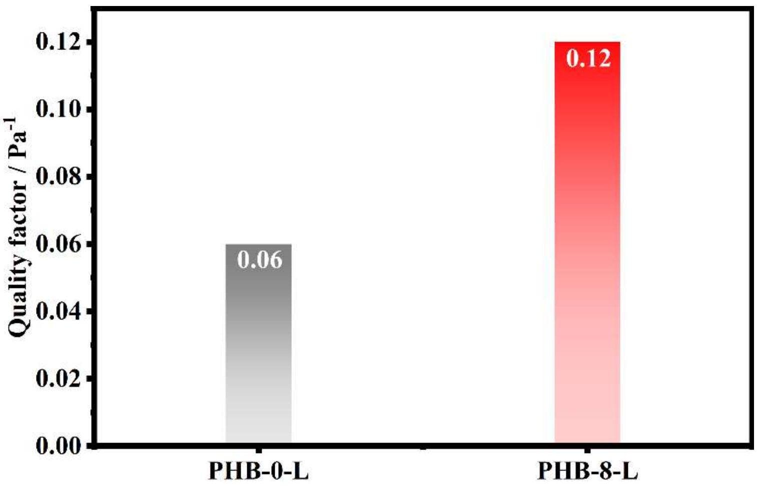

Figure S9. A comparison of the quality factors of PHB-0-L (without barium titanate) and PHB-8-L.

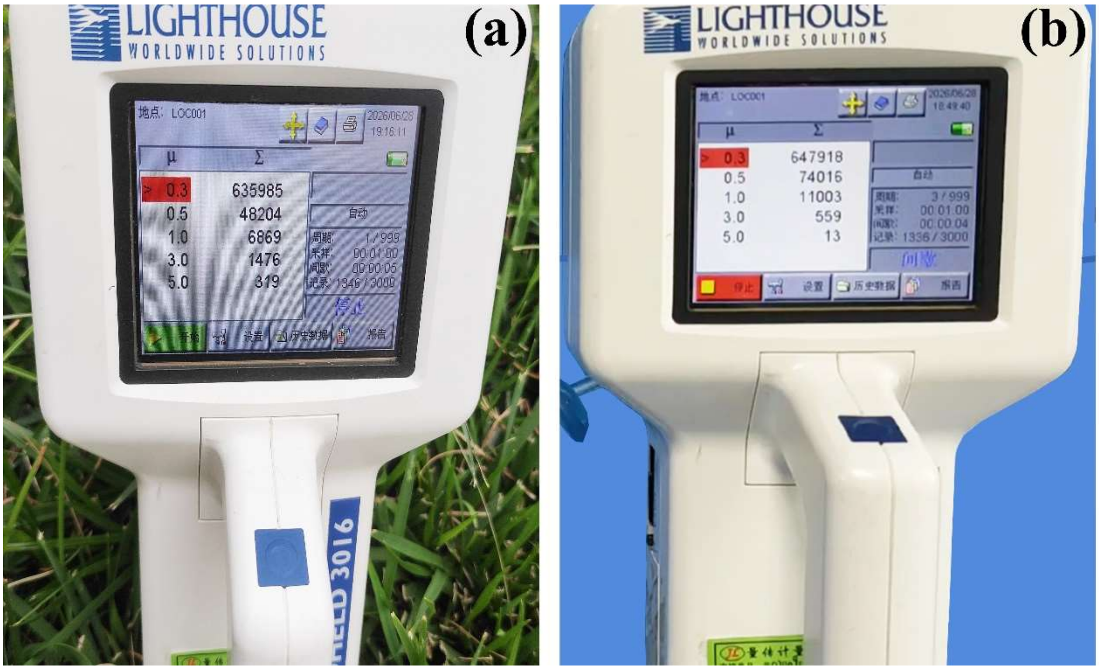

Figure S10. Photographs showing particulate matter concentrations before and after filtration of smoke using the PHB-8-L: (a) Before filtering; (b) After filtering.

Text S1. The electrostatic field distribution of the receiver plates

The electrostatic field distribution of the needle-back electrode system was investigated through finite element analysis using the COMSOL Multiphysics®

software package, specifically its AC/DC Module with the Electrostatics interface. The computational model consisted of a vertical stack of a suspended needle electrode, an air gap, and a conductive substrate. Three substrate configurations were examined: (1) a hexagonal porous conductive plate with a pore side length of 4 mm, (2) a hexagonal porous conductive plate with a pore side length of 2 mm, and (3) a solid conductive plate. A large air domain surrounding this core structure was included to approximate an unbounded space. A three-dimensional axisymmetric model was developed to efficiently compute the fields with high resolution. The conductive substrate was defined as a horizontal block with a constant potential boundary condition -4 kV). In the porous configurations, the solid portions were treated as perfect conductors, and the pores were filled with air ($\varepsilon_r = 1.0$). The suspended needle electrode, positioned above the substrate with a separation distance of 20 cm, was modeled with a finite tip curvature and assigned an electric potential of +22 kV to establish a high voltage difference with the grounded substrate. The surrounding environment was modeled as air ($\varepsilon_r = 1.0$). The governing equation for the electrostatic field is Poisson's equation: $\nabla\cdot(\varepsilon\nabla V) = 0$, where V is the electric potential and ε is the permittivity of the material. The electric field E was subsequently derived from the computed potential as $E = -\nabla V$. The outer boundaries of the surrounding air domain were set to a "Zero Charge" condition ($n\cdot D = 0$), which simulates a condition where the electric field lines are parallel to the boundary, effectively representing a domain open to infinity. A physics-controlled mesh was employed with substantial local refinement applied around the needle tip and the substrate–air interface (including the edges of the pores for the porous

plates), where the highest electric field gradients were anticipated. For each group, the stationary solver computed the resultant electric potential and electric field distributions.

Text S2. The specific test procedure of the bubble point method

The specific test procedure involved thoroughly wetting the membrane to be tested with a wetting liquid and then mounting it in the instrument's fixture for testing. Due to surface tension, the wetting liquid is retained within the membrane's pores. Gradually increasing gas pressure is applied to one side of the membrane. When the gas pressure exceeds the surface tension of the wetting solution within a particular pore, the wetting solution in that pore is displaced by the gas. The pressure at which the first pore opens is termed the bubble point pressure, and the pore size corresponding to this pressure is the maximum pore size. During the test, pressure and flow rate are recorded in real time to produce a pressure-flow rate relationship curve. The maximum, average and minimum pore sizes, as well as the pore size distribution, can then be calculated using the relevant formulae.
